# WHO Mental Health Gap Action Programme Intervention Guide (mhGAP-IG): the first pre-service training study

**DOI:** 10.1186/s13033-020-00379-2

**Published:** 2020-06-29

**Authors:** Ashmita Chaulagain, Laura Pacione, Jibril Abdulmalik, Peter Hughes, Kopchak Oksana, Stanislav Chumak, José Mendoza, Kristine Avetisyan, Gayane Ghazaryan, Khachatur Gasparyan, Eka Chkonia, Chiara Servili, Neerja Chowdhury, Iryna Pinchuk, Myron Belfar, Anthony Guerrero, Lilya Panteleeva, Norbert Skokauskas

**Affiliations:** 1 Regional Centre for Child and Youth Mental Health and Child Welfare, Norwegian University of Science and Technology (NTNU), Trondheim, Norway; 2 Department of Psychiatry, Division of Child and Youth Mental Health, University of Toronto, Toronto, Canada; 3 Department of Psychiatry, University of Ibadan, Ibadan, Nigeria; 4 Institute of Psychiatry, Psychology and Neuroscience, King’s College London, London, UK; 5 Department of Neurology, Psychiatry, Kyiv Medical University, Kiev, Ukraine; 6 Department of Psychiatry, Psychotherapy and Medical Psychology, P.L. Shupyk National Medical Academy of Postgraduate Education, Kiev, Ukraine; 7 Department of Psychiatry, National Autonomous University of Mexico, Mexico City, Mexico; 8 Department of Clinical Psychology, Yerevan State Medical University, Yerevan, Armenia; 9 Department of Psychiatry, Tbilisi State Medical University, Tbilisi, Georgia; 10 Department of Mental Health and Substance Abuse, World Health Organization, Geneva, Switzerland; 11 Research Institute of Psychiatry and Drug Abuse, Ministry of Health, Kiev, Ukraine; 12 Department of Psychiatry, Harvard Medical School, Boston, USA; 13 Child and Adolescent Psychiatry Division, University of Hawai’i at Mānoa John A. Burns School of Medicine, Honolulu, USA; 14 Department of Psychiatry, Kyrgyz-Russian Slavic University, Bishkek, Kyrgyzstan; 15 Helse Midt-Norge RHF, Trondheim, Norway

**Keywords:** World Health Organization, Mental Health Gap Action Programme (mhGAP), Mental Health Gap Action Programme Intervention Guide (mhGAP-IG), Pre-service training, Medical education, Nursing education, Mental disorders, Treatment gap

## Abstract

**Background:**

Despite the increasing burden of mental, neurological, and substance use (MNS) disorders, a significant treatment gap for these disorders continues to exist across the world, and especially in low- and middle-income countries. To bridge the treatment gap, the World Health Organization developed and launched the Mental Health Gap Action Programme (mhGAP) and the mhGAP Intervention Guide (mhGAP-IG) to help train non-specialists to deliver care. Although the mhGAP-IG has been used in more than 100 countries for in-service training, its implementation in pre-service training, that is, training prior to entering caregiver roles, is very limited.

**Aim of the study:**

The aim of this study was to collect and present information about the global experience of academic institutions that have integrated WHO’s mhGAP-IG into pre-service training.

**Methods:**

A descriptive cross-sectional study was conducted using an electronic questionnaire, from December 2018 to June 2019.

**Results:**

Altogether, eleven academic institutions across nine countries (Mexico, Nigeria, Liberia, Sierra Leone, Somaliland, Armenia, Georgia, Ukraine and Kyrgyzstan) participated in this study. Five of the institutions have introduced the mhGAP-IG by revising existing curricula, three by developing new training programmes, and three have used both approaches. A lack of financial resources, a lack of support from institutional leadership, and resistance from some faculty members were the main obstacles to introducing this programme. Most of the institutions have used the mhGAP-IG to train medical students, while some have used it to train medical interns and residents (in neurology or family medicine) and nursing students. Use of the mhGAP-IG in pre-service training has led to improved knowledge and skills to manage mental health conditions. A majority of students and teaching instructors were highly satisfied with the mhGAP-IG.

**Conclusions:**

This study, for the first time, has collected evidence about the use of WHO’s mhGAP-IG in pre-service training in several countries. It demonstrates that the mhGAP-IG can be successfully implemented to train a future cadre of medical doctors and health nurses.

## Background

### Global prevalence of mental, neurological, and substance use disorders

Mental, neurological, and substance use (MNS) disorders are highly prevalent globally, affecting people in every community and age group across all regions of the world [1, 2]. According to the World Health Organization (WHO), one in four people globally will be affected by a mental or neurological disorder at some point during their lives [3].

Mental and addictive disorders affected more than 1 billion people globally in 2016 [4]. These disorders affected women and men to a similar degree: 537,698,000 women (uncertainty interval 545,451,000–598,555,000) and 572,376,000 men (uncertainty interval 513,713,000–562,164,000). MNS disorders caused 7% of all global burden of disease as measured in disability-adjusted life years (DALYs) and 19% of all years lived with disability. Depression was associated with most DALYs for both sexes, with higher rates in women as all other internalizing disorders, whereas other disorders such as those associated with substance use had higher rates in men [4].

### Treatment gap for MNS disorders

While mental and addictive disorders affect more than 1 billion people globally, there is a huge gap between the need for services and the provision of services worldwide [1, 5]. The treatment gap for persons with MNS disorders exceeds 50% in all countries of the world, and approaches astoundingly high rates of 90% in the least resourced countries, even for serious disorders associated with significant functional impairments and risk of death [6]. While at least one in 10 people suffering from a mental health problem at any one time, only 1% of the global health workforce provides mental health care [5]. According to the 2018 WHO Mental Health Atlas, there is only one psychiatrist per 100,000 people in over half the countries in the world [7]. The absolute number of mental health care workers per 100,000 population varies immensely; for instance, there are on average 11.9 psychiatrists per 100,000 population in high-income countries compared with fewer than 0.1 in low-income countries. Similarly, there are 23.5 nurses providing mental health care per 100,000 population in high-income countries compared with 0.3 nurses in low-income countries, 1.4 in lower-middle-income countries, and 6.8 in upper-middle-income countries [7]. There are even fewer child psychiatrists, with fewer than 0.1 per 100,000 population in all countries except the high-income group, where the number of child psychiatrists is 1.19.

### Reducing the treatment gap for MNS disorders

The treatment gap for MNS disorders is well documented [8–10], and to reduce it WHO has developed the Mental Health Gap Action Programme (mhGAP) [1] and the Mental Health Gap Action Programme Intervention Guide (mhGAP-IG) [11]. mhGAP outlines key steps for scaling up mental health services in low- and middle-income countries (LMICs) [1], while the mhGAP-IG provides evidence-based guidelines and tools for the assessment and integrated management of priority MNS disorders in non-specialized health settings [11].

WHO’s mhGAP-IG has been used for in-service training to build the capacity of non-specialized health care providers to assess and manage priority MNS disorders in more than 100 countries, and it has been translated into more than 20 languages [5]. Apart from in-service training, another approach to reduce the treatment gap in MNS disorders is through using the mhGAP-IG in pre-service training. This refers to the introduction of mhGAP-IG materials, concepts, and approaches in teaching programmes for health workers before they enter service roles [12]. However, a recent systematic review of the mhGAP-IG underscored that it continues to be used mainly in in-service training for the existing health care workforce and its implementation in pre-service training remains very limited (or is not reported). According to this review, only one university reported using the mhGAP-IG in pre-service training [5].

While mhGAP-IG in-service training is important in building the capacity of health care professionals in non-specialized health settings to assess and manage priority MNS disorders, there are long-term advantages also to integrating the mhGAP-IG into pre-service training. These include: i) sustainability, as the mhGAP-IG can be integrated into teaching curricula, does not require additional courses, and is periodically updated by WHO based on current evidence; ii) cost-effectiveness, as it reduces the need for more expensive in-service training; iii) the opportunity to develop a common understanding among different categories of health workers about the assessment and management of common MNS disorders; iv) strengthening of health systems in the long term by building the capacity of the health workforce to provide high-quality evidence-based care [12]; and providing a reference point for later in-service training. Integrating the mhGAP-IG into pre-service training is therefore an important public health opportunity. To facilitate its uptake, it is important to collect information from institutions that have already implemented mhGAP-IG materials into their pre-service training courses to understand the benefits, and the challenges.

### Aims and specific objectives of the study

The aim of this study was to collect data on the experiences of academic institutions worldwide that have integrated the mhGAP-IG into pre-service training.

### Specific objectives

The specific objectives of this study were:

1. To gather information about the preparation and adaptation phases of mhGAP-IG pre-service training at academic institutions (including the initial decision to use the mhGAP-IG, financial support, piloting of the mhGAP-IG, obstacles to its introduction, etc.);
2. to collect information about learners and teaching processes in mhGAP-IG integration in pre-service training (i.e., academic background of trainees, mhGAP-IG modules used, types of resources, methods used to teach the mhGAP-IG, total duration of mhGAP-IG pre-service training, etc.);
3. to present information about the outcomes of mhGAP-IG pre-service training (including the number of students who benefited, facilitative and challenging factors to mhGAP-IG implementation, feedback from students, clinical instructors, and other stakeholders);
4. to gather recommendations from representatives of the institutions that have implemented the mhGAP-IG.

## Methodology

### Study settings

In 2018, three consultative meetings took place on WHO mhGAP-IG implementation in pre-service training:

i. a consultation on mhGAP pre-service training in child and adolescent mental health in Prague, Czech Republic on July 25, during the 23rd World Congress of the International Association for Child and Adolescent Psychiatry and Allied Professions (IACAPAP);
ii. a WHO Informal Consultation on mhGAP pre-service training in general mental health in Mexico City, Mexico on September 29, during the 18th World Psychiatric Association (WPA) World Congress of Psychiatry;
iii. a parallel session on the mhGAP-IG and pre-service training at the 2018 mhGAP Forum held at WHO’s headquarters in Geneva, Switzerland. The 2018 mhGAP Forum took place on October 11–12 and provided an opportunity for diverse stakeholders to discuss progress on WHO’s Mental Health Action Plan 2013–2020. The theme of the forum was “Accelerating Country Action on Mental Health”, which reflected the vision of WHO’s 13th General Programme of Work. The forum hosted the mhGAP pre-service consultation meeting on October 12, 2018.

These consultative meetings acted as an initial platform and stimulus for the first mhGAP pre-service training study. The events facilitated coordination with educators who had experience of introducing and implementing the mhGAP-IG in pre-service training at their own institutions. These medical educators were later contacted via email and invited to participate in the study.

### Study design

A cross-sectional descriptive study was conducted, using an electronic questionnaire, from December 2018 to June 2019.

### Study area and sample

Academic institutions in North America, Africa, Eastern Europe, and Central Asia were included. Twelve institutions were initially selected through convenience sampling to participate in the study. Eleven responded to our questionnaire.

### Data collection tools

A self-administered electronic questionnaire was developed that consisted of 32 questions divided into four sections: (i) background (i.e., basic information about the institution and the respondent’s role there); (ii) preparation and adaptation (i.e., reasons for using the mhGAP-IG, financial support, adaptation, piloting, obstacles, mode of integration, trainers, etc.); (iii) teaching process (i.e., type of trainees and faculty members involved, mhGAP-IG modules used, resources and methods use to teach the mhGAP-IG, duration of training, etc.); and (iv) outcomes (i.e., total number of students trained, identified benefits, facilitating factors and challenges of using the mhGAP-IG in pre-service training, etc.). The data collection instrument included both closed-ended and open-ended questions.

### Pilot testing

The questionnaire was reviewed and refined by an expert panel with experience in the mhGAP-IG.

### Data analysis

Descriptive data are presented in tabulated form to provide information about the background, preparation and adaptation phase, teaching process, and outcomes of the academic institutions.

### Ethical approval

The purpose of this study was to gather publicly available information about mhGAP-IG integration, and the person who completed the survey was not the focus of the research. Implicit consent was provided when respondents completed the questionnaire.

The academic institutions surveyed were: the National Autonomous University of Mexico, Mexico City, Mexico; University of Ibadan, Ibadan, Nigeria; Phebe Paramedical Training Program and School of Nursing, Suakako, Bong, Liberia; University of Hargeisa, Hargeisa, Somaliland; Amoud University, Borama, Somaliland; Sierra Leone Medical School, Freetown, Sierra Leone; P.L. Shupyk National Medical Academy of Postgraduate Education, Kyiv, Ukraine; Kyiv Medical University, Kyiv, Ukraine; Tbilisi State Medical University, Tbilisi, Georgia; Yerevan State Medical University, Yerevan, Armenia; and Kyrgyz-Russian Slavic University, Bishkek, Kyrgyzstan.

One respondent completed questionnaires for the University of Hargeisa, Somaliland; Amoud University, Somaliland; and Sierra Leone Medical School, Sierra Leone, as the same person was responsible for both implementing and teaching the mhGAP-IG at these institutions. Eight respondents were psychiatrists, one was a clinical psychologist, and one was a psychiatric nurse. The respondents were responsible for teaching medical or nursing students or doctors in training at their respective institutions.

## Results

Below we provide information about the experience of each academic institution with its implementation of the mhGAP-IG, including preparation activities, obstacles, teaching processes, supporting factors, and benefits and challenges (Table 1).

**Table 1:** The mhGAP-IG in pre-service training: introduction, implementation, and outcomes

Institution	Preparation phase							Teaching process				
	mhGAP-IG introduction	Reasons for using mhGAP-IG	Alternatives considered to mhGAP-IG	Language	mhGAP-IG translated	Approaches taken to integrate mhGAP-IG into existing curriculum	Funding	mhGAP-IG modules used in teaching	Teaching–Instructors	Teaching –format	Duration of mhGAP-IG training	Number of students trained
Universidad Nacional Autónoma de México, Mexico City, Mexico	Revision of the existing curriculum	mhGAP-IG content; huge treatment gap	None	Spanish	No^a^	Allocated time for mhGAP-IG in lectures and removed other elements from the curriculum	None	All 10 modules	Assistant professor of psychiatry	Didactic teaching settings (in lecture hall) and in small group settings	20 h	50 medical doctors
University of Ibadan, Ibadan, Nigeria	Revision of the existing curriculum	Evidence-based; freely available; decision informed by local survey/research on competencies and training needs; mhGAP-IG content; and huge treatment gap	None	English	No	Allocated time for mhGAP-IG in lectures; removed other elements from the curriculum; and utilized mhGAP-IG as a clinical tool during placements	None	Psychosis; depression; drug and alcohol use disorders; behavioral disorders; and developmental disorders	Professor of psychiatry	Didactic teaching and role-play	Six hours for medical students and 5 days for master’s students	455 medical students and 95 master’s students
Phebe Paramedical Training Program and School of Nursing, Suakako, Bong, Liberia	Revision of the existing curriculum	Evidence-based; decision informed by stakeholder consultation and mhGAP-IG content	None	English	No	Allocated time for mhGAP-IG in lectures; removed other elements from the curriculum; and utilized mhGAP-IG as a clinical tool during placements	Carter Center Mental Health Program and the Government of Liberia	Behavioral disorder management	Clinical instructors	Didactic teaching, small group teaching, and as a clinical placement tool	4 months	200 nurses
University of Hargeisa, Hargeisa, Somaliland	New training program	Evidence-based; freely available; decision informed by stakeholder consultation; mhGAP-IG content; and huge treatment gap	Traditional psychiatric curriculum	English	No	N/A	None	All mhGAP-IG modules except module on alcohol and substance use disorders	Professor of psychiatry; clinical Instructors	Didactic teaching; role-play and small group teaching, along with real clinical contacts	11 days for medical students and 10 days for nursing students	320 medical students and 30 nurses
Sierra Leone Medical School, Freetown, Sierra Leone	New training program	Evidence-based; freely available; decision informed by stakeholder consultation; mhGAP-IG content; and huge treatment gap	Traditional psychiatric curriculum	English	No	N/A	None	All 10 modules	Professor of psychiatry; clinical Instructors; tutors;	Didactic teaching; role-play and small group teaching along with real clinical contacts	1 week	40 medical students
University of Amoud, Borama, Somaliland	New training program	Evidence-based; freely available; decision informed by stakeholder consultation; mhGAP-IG content; and huge treatment gap	Traditional psychiatric curriculum	English	No	N/A	None	All mhGAP-IG modules except module on alcohol and substance use disorders	Professor of psychiatry; clinical Instructors	Didactic teaching; role-play and small group teaching, along with real clinical contacts	11 days	160 medical students
P.L. Shupyk National Medical Academy of Postgraduate Education, Kyiv, Ukraine	Revision of the existing curriculum	Evidence-based; freely available; and mhGAP-IG content	None	Russian	Yes	Allocated time for mhGAP-IG in lectures and removed other elements from the curriculum	None	Depression module	Lecturer in psychiatry	Didactic teaching	2 days	30 doctors in training
Kyiv Medical University, Kyiv, Ukraine	Revision of the existing curriculum	Evidence-based and freely available	None	Ukrainian and English (for international students)	Yes	Allocated time for mhGAP-IG in lectures; removed other elements from the curriculum; utilized mhGAP-IG as a clinical tool during placements; and small group learning activity	None	Depression; dementia; epilepsy; behavioral disorders	Professor of neurology; professor of psychiatry; and tutors	Didactic teaching in lecture group learning; and as a clinical placement tool	2 weeks	30 medical students
Tbilisi State Medical University, Tbilisi, Georgia	Revision of the existing curriculum	Evidence-based; freely available; and huge treatment gap	None	Georgian	Yes	Allocated time for mhGAP-IG in lectures and removed other elements from the curriculum; utilized mhGAP-IG as a clinical tool during placements; and small group learning activity	None	Depression; dementia; bipolar disorder; suicide; self-harm; anxiety disorder	Professor of psychiatry	Introduced as a clinical tool during placements	1 week	16 medical students^b^
Yerevan State Medical University after Mkhitar Heratsi, Yerevan, Armenia	Revision of the existing curriculum	mhGAP-IG content; huge treatment gap	None	Armenian	Yes	Allocated time for mhGAP-IG in lectures and removed other elements from the curriculum; utilized mhGAP-IG as a clinical tool during placements; and small group learning activity	None	Depression; ADHD; suicide	Associate professors of clinical psychology	Didactic teaching in lecture format and small group learning	8 h	480 medical students
Kyrgyz-Russian Slavic University named after B.N. Yeltsin, Bishkek, Kyrgyzstan	Revision of the existing curriculum	Evidence-based; freely available; mhGAP-IG content; and huge treatment gap	None	Russian/Kyrgyz	Yes	Allocated time for mhGAP-IG in lectures and removed other elements from the curriculum; utilized mhGAP-IG as a clinical tool during placements	Not received	Depression; dementia; suicide; substance abuse; and child and adolescent mental and behavioral disorders	Professors/researchers in psychiatry	Didactic teaching and small group learning	6 months	12 medical students

Institution	Outcomes				
	Knowledge testing	Students’ response to mhGAP-IG	Teaching instructors’ response to mhGAP-IG	Clinical administrators’ response to mhGAP-IG	Recommendations made by respondents
Universidad Nacional Autónoma de México, Mexico City, Mexico	Post-exams	Highly satisfied (HS)	HS	HS	Use small groups and short courses; include students in the training work
University of Ibadan, Ibadan, Nigeria	Pre- and post-tests; exams	HS	HS	Satisfied (S)	Increase duration of training and use outcome assessment
Phebe Paramedical Training Program and School of Nursing, Suakako, Bong, Liberia	Pre- and post-tests; students’ presentations	HS	S	HS	Have two-way communication between students and instructors about training modules
University of Hargeisa, Hargeisa, Somaliland	Pre- and post-tests; clinical OSCE (objective clinical structured exam)and observed role-plays	HS	S	S	Ensure that psychiatrists accept mhGAP-IG; ensure support at the policy level; and provide training to local educators
Sierra Leone Medical School, Freetown, Sierra Leone	Pre- and post-tests; clinical OSCEs; and observed role-plays	HS	S	S	Ensure psychiatrists’ buy-in and adopt public health approach; ensure support from ministry level; and more local champions
University of Amoud, Borama, Somaliland	Pre- and post-tests; clinical OSCEs; and observed role-plays	HS	S	S	Ensure that psychiatrists move from traditional teaching to use of mhGAP-IG; ensure support at the policy level; and provide training to local educators
P.L. Shupyk National Medical Academy of Postgraduate Education, Kyiv, Ukraine	Pre- and post-tests	HS	HS	–	More emphasis on feedback from students and assessment of their knowledge
Kyiv Medical University, Kyiv, Ukraine	Pre- and post-tests	HS	S	S	–
Tbilisi State Medical University, Tbilisi, Georgia	Case discussion and multiple choice questions (MCQs)	Satisfied (S)	S	HS	–
Yerevan State Medical University after Mkhitar Heratsi, Yerevan, Armenia	Exam and practical session	HS	HS	HS	–
Kyrgyz-Russian Slavic University named after B.N. Yeltsin, Bishkek, Kyrgyzstan	MCQs; pre- and post-training assessment	HS	S	S	Conduct informational trainings and meetings with representatives of the Ministry of Education in the presence of the WHO Country Office

^a^UNAM used WHO’s translated Spanish version of the mhGAP-IG

^b^TSMU trained four students in each psychiatric clerkship program

### National Autonomous University of Mexico, Mexico

Mexico is a country in the southern portion of North America with a total population of 123.5 million [13]. It is an upper-middle-income country, with a poverty rate of 26.2% [14]. The National Autonomous University of Mexico (UNAM) was founded in 1551 and is the oldest university in North America, and its school of medicine is one of the oldest in the Americas [15]. In 2018, UNAM School of Medicine started using an mhGAP-IG-based curriculum in its undergraduate programme.

#### Preparation and adaptation phase

The UNAM School of Medicine took 6 months to adapt the mhGAP-IG training materials. It piloted them with medical students, who were well satisfied and even wanted more time for mhGAP-IG training. Therefore, the university created a series of five-hour sessions (compared with an initial three hours) spread over 4 days. A lack of finance and a lack of trained instructors were reported as the main obstacles to introducing the mhGAP-IG into pre-service training.

#### Teaching process

UNAM currently trains medical students with the mhGAP-IG using resources such as the training package with videos and role-plays; the mhGAP-IG app for smartphones and tablets; and custom teaching tools created by the faculty itself (e.g., handouts, presentations, etc.) for training.

#### Outcomes

The willingness of the university to implement the mhGAP-IG and its supportiveness were mentioned as facilitating factors, while a lack of time to provide training to students and for evaluation were reported as the main challenges. However, the mhGAP-IG pre-service training has led to better knowledge about the assessment and management of mental disorders.

### University of Ibadan, Nigeria

Nigeria, with 186 million inhabitants, is the most populous country in Africa and the seventh most populous in the world [16]. It is a lower-middle-income country, with 50% of its population living below the poverty line [14].

Established in 1948, the University of Ibadan (UI) was the first university in Nigeria, and the Faculty of Medicine was one of the first faculties to be created. It is widely regarded as a center of excellence in West Africa for clinical medicine, training of health care professionals, and medical research [17]. The faculty integrated the mhGAP-IG into its curriculum in 2015.

#### Preparation and adaptation phase

UI spent 12 months planning, adapting, and integrating the mhGAP-IG training package into the medical school’s curriculum. No obstacles were reported to integrating the mhGAP-IG into pre-service training.

#### Teaching process

UI trains medical students and master’s degree students in child and adolescent mental health using the mhGAP-IG. For training it uses the print version of the mhGAP-IG, along with the mhGAP-IG training package, which includes videos and role-plays, and the mhGAP-IG app.

#### Outcomes

Institutional support was reported as the main factor that helped to implement mhGAP-IG training, with insufficient time to train future medical doctors cited as the main difficulty faced when using it. The identified advantages of mhGAP-IG pre-service training were improved attitude among students and confidence to handle mental health conditions.

### Phebe Paramedical Training Program and School of Nursing, Liberia

Liberia is a country in West Africa with a population of about 4.5 million. It is one of the poorest countries in the world, with a poverty rate of 54% [18].

The Phebe Paramedical Training Program and School of Nursing is the first and largest paramedical and nursing school in the country [19]. It introduced the mhGAP-IG into its curriculum in 2017.

#### Preparation and adaptation phase

Phebe Paramedical Training Program and School of Nursing took 10 months to adapt the mhGAP-IG content and to create a 4-month curriculum. It piloted this with students as part of its mental health course and collected feedback; students found the mhGAP-IG very useful. Limited time and trainees having difficulty in understanding some of the mhGAP-IG topics were reported as the most significant barriers slowing down the introduction of the mhGAP-IG into pre-service training.

#### Teaching process

The mhGAP-IG is taught to nursing students using the training package, which comprises videos and role-plays; the mhGAP-IG printed version; and custom teaching tools created by the institution itself (e.g., handouts, presentations, etc.).

#### Outcome

The identified benefits of mhGAP-IG pre-service training were ease of use, comprehensibility, and enhanced skills and knowledge. No facilitating factors or challenges were reported.

### Sierra Leone Medical School, Sierra Leone

Sierra Leone is a country in West Africa with a population of 7,075,641. It is one of the world’s poorest countries, with more than 60% of the population living on less than US$1.25 a day [20].

Its first and so far only medical school (The College of Medicine and Allied Health Sciences) was established in 1988 by the Government of Sierra Leone with the Nigerian government and WHO [21]. The Medical School introduced the mhGAP-IG into its curriculum in 2016.

#### Preparation and adaptation phase

The Sierra Leone Medical School integrated the mhGAP-IG into its curriculum without adaptation. The absence of assistance from institutional management and opposition from within the faculty were noted as the main barriers to introducing the mhGAP-IG.

#### Teaching process

For training medical students, the institution uses the mhGAP-IG training package, which includes role-plays and videos, and the printed version of the mhGAP-IG.

#### Outcomes

Students’ acquisition of better mental health skills in all clinical areas was reported as the main advantage of the mhGAP-IG pre-service training. Similarly, support from the university, including its enthusiastic and public health-minded faculty, was identified as an enabling factor for the introduction and implementation of the mhGAP-IG in pre-service training. However, resistance from some psychiatrists to teaching and a lack of time for pre-service training were mentioned as challenges faced while using the mhGAP-IG.

### University of Hargeisa, Somaliland

Somaliland is a self-declared state, considered internationally to be an autonomous region of Somalia. It lies in northwestern Somalia and has a population of approximately four million. It is one of the poorest countries in the world, with a poverty rate of 73% [22].

The University of Hargeisa (UOH) was established in 2000 and is the largest and leading chartered public university in Somaliland [23]. In 2011, UOH’s College of Medicine & Health Sciences introduced the mhGAP-IG into its curriculum.

#### Preparation and adaptation phase

UOH faculty members and volunteers from the UK took 2 weeks to adapt the mhGAP-IG for use in the curriculum. UOH made some adaptations on the etiology of mental disorders and skills that are more clinical in nature, and cultural adaptations. In Somaliland there is a common cultural belief that djinn (spirits) may be the cause of mental health problems, especially if hallucinations are present, so UOH made cultural adaptations regarding how to address this notion from a multicultural perspective. However, it did not pilot the adapted mhGAP-IG curriculum. Insufficient support from the institution’s leadership and resistance from some faculty members were reported as significant barriers to introducing the mhGAP-IG into pre-service training.

#### Teaching process

Medical and nursing students at UOH are being trained using presentations, videos and role-plays, and the mhGAP-IG print version.

#### Outcomes

While better clinical skills were identified as a benefit of the mhGAP-IG pre-service training, opposition from some psychiatrists, a shortage of time, and training students through real clinical contacts were reported as challenges encountered in its use. However, the university’s receptivenessn to the mhGAP-IG and the presence of active team members facilitated its integration into pre-service training.

### Amoud University, Somaliland

Amoud University (AU), is the oldest and most comprehensive public university in Somaliland, founded in 1997. Amoud College of Health Sciences is the fastest-growing institute in the university. It introduced the mhGAP-IG into the curriculum in 2011.

#### Preparation and adaptation phase

AU, in collaboration with UOH and volunteers from the UK, took 2 weeks to adapt the mhGAP-IG for use in the curriculum. It also made some adaptations on the etiology of mental disorders, clinical skills, and cultural considerations. Like UOH, it did not pilot the adapted mhGAP-IG curriculum prior to introducing it. Lack of support from institutional leadership and resistance from some faculty members were reported as the major obstacles to introducing the mhGAP-IG into pre-service training.

#### Teaching process

The institution used the mhGAP-IG print version, together with videos, role-plays, and presentations to train future medical doctors.

#### Outcomes

Resistance by some local mental health staff to change and to moving away from a didactic teaching approach, and managing the time for clinical-based contact and lecture hall-based sessions, were reported as challenges faced during implementation of the mhGAP-IG. Openness to its use and supportiveness from the university and from psychiatrists were mentioned as enabling factors in its implementation. Improved skills to identify and manage mental disorders were identified as a benefit of the mhGAP-IG pre-service training.

### Shupyk National Medical Academy of Postgraduate Education, Ukraine

Ukraine is a country in Eastern Europe with a population of around 42.5 million [24]. It is a lower-middle-income country, with 24.1% of the population living below the poverty line [14].

The P.L. Shupyk National Medical Academy of Postgraduate Education (NMAPE) was one of the first academic and research institutions in Ukraine, founded in 1918 during the period of the Ukrainian State at a time of revival for Ukrainian education, science, and technology [25]. It introduced the mhGAP-IG into its curriculum in early 2019.

#### Preparation and adaptation phase

NMAPE took 1 month to adapt some of the management parts of the mhGAP-IG module on depression. The institution piloted the depression module with its students and gathered feedback; students expressed an interest in the module. A lack of finance was reported as the most significant deterrent to introducing the mhGAP-IG into pre-service training.

#### Teaching process

At NMAPE, medical residents in neurology and medical psychology are being trained with resources that include the mhGAP-IG training package, which comprises videos and role-plays; they also use modified presentations.

#### Outcomes

Willingness by the department to implement the mhGAP-IG was cited as the factor that most helped with its incorporation into pre-service training, and well-structured information with visual materials was identified as its main advantage. The unavailability of the mhGAP-IG in a Ukrainian-language version was reported as the main challenge. NMAPE did not use the English or Russian versions, but translated the depression module into Ukrainian itself.

### Kyiv Medical University, Ukraine

Kyiv Medical University (KMU) is the largest private medical university in Ukraine, and was founded in 1992 [26]. It introduced the mhGAP-IG into its curriculum in December 2018.

#### Preparation and adaptation phase

KMU did not adapt the mhGAP-IG; it piloted it in the curriculum with neurology residents, and received positive feedback. No obstacles were reported to introducing it into pre-service training.

#### Teaching process

KMU uses a training package that includes presentations, videos and role-plays, the mhGAP-IG print version, and custom teaching tools created by the faculty (e.g., handouts, presentations, lectures) to provide training to medical residents in neurology.

#### Outcomes

The main benefit of the new teaching approach has been trainees’ improved knowledge about mental health disorders. Support from the university administration was mentioned as an enabling factor in implementing the mhGAP-IG in pre-service training, while the lack of a Ukrainian-language version of the guide was identified as the major challenge encountered.

### Tbilisi State Medical University, Georgia

Georgia is located at the crossroads of Western Asia and Eastern Europe. It has a population of 3.718 million and is a lower-middle-income country, with 22% of the population living below the poverty line [27].

Tbilisi State Medical University (TSMU) is a leading medical university [28]. It began using the mhGAP-IG in its curriculum in 2018.

#### Preparation and adaptation phase

TSMU took 1 week to adapt the mhGAP-IG. A limited support from leaders of the institution and resistance from faculty members were recognized as significant barriers to incorporating it into pre-service training.

#### Teaching process

The print version of the mhGAP-IG is used to provide training to medical students.

#### Outcomes

No challenges were identified during the implementation of the mhGAP-IG. Additionally, assistance from the university leadership was reported as the main facilitating factor, while the identified advantage of the mhGAP-IG in pre-service training was that it was easy to use and understand.

### Yerevan State Medical University, Armenia

Armenia is located in Eastern Europe and has a population of 2.93 million [29]. It is an upper-middle-income country, with 29.8% of its population living below the poverty line [30].

Yerevan State Medical University after Mkhitar Heratsi (YSMU) is the country’s leading medical university, and was founded in 1920 [31]. It introduced the mhGAP-IG into its curriculum in early 2019.

#### Preparation and adaptation phase

YSMU took 1 month to adapt the mhGAP-IG. It piloted the mhGAP-IG-based curriculum with medical students and received positive feedback. A lack of financial resources and lack of support from decision makers were identified as the most significant obstacles to introducing the mhGAP-IG into pre-service training.

#### Teaching process

Future medical doctors are trained using the mhGAP-IG print version and handouts, presentations, lectures, etc. prepared by the faculty.

#### Outcomes

The mhGAP training of trainers (ToT) course organized by UNA Partnership in 2018 and students’ interest in the mhGAP-IG were reported as factors that enabled its introduction and implementation. The main reported advantage of the mhGAP-IG pre-service training was the acquisition of better knowledge and skills to identify and manage mental disorders.

### Kyrgyz-Russian Slavic University, Kyrgyzstan

Kyrgyzstan is a landlocked country in Central Asia with a population of 6.4 million. It has the second lowest gross national income (GNI) in the region, and 32% of the population live below the poverty line [32].

The Kyrgyz-Russian Slavic University (KRSU) was founded in 1993 under the Warsaw Treaty of Friendship, Cooperation and Mutual Assistance between the Kyrgyz Republic and the Russian Federation [33]. The institution began pre-service training based on the mhGAP-IG in early 2019.

#### Preparation and adaptation phase

It took around 5 months for KRSU to adapt the mhGAP-IG. It piloted a curriculum based on it with trainee general practitioners and received positive feedback. Financial obstacles and a lack of support from institutional leaders were reported as being among the major obstacles to introducing the mhGAP-IG into pre-service training.

#### Teaching process

The institution created a training package that includes presentations, videos and role-plays, the mhGAP-IG print version, and custom teaching tools created by the faculty (e.g., handouts, presentations, lectures) to train medical residents.

#### Outcomes

A positive response from the university administration was cited as a supporting factor for the implementation of the mhGAP-IG. The identified benefit of the mhGAP-IG was an increase in students’ knowledge and skills relating to mental disorders and their management. The “traditional teaching practices” of academic staff were reported as a challenge encountered with use of the mhGAP-IG in pre-service training.

## Discussion

### Interpretation of the main findings

This study has, for the first time, collected data about the introduction and use of WHO’s mhGAP-IG in pre-service training settings globally. The mhGAP-IG has been successfully used in different regions of the world and in very different academic institutions. For example, it has been successfully introduced in the oldest and most traditional national medical schools in some countries (e.g., UNAM in Mexico and UI in Nigeria) and also in newly established public (e,g., UOH in Somaliland) and private medical schools (e.g., KMU in Ukraine). This demonstrates the universality, adaptability, and acceptability of the mhGAP-IG in pre-service teaching. In addition, different studies [35] have highlighted the importance of the psychiatric community accepting the concept of the mhGAP-IG in order for it to be successfully implemented. In this study the majority of the respondents were psychiatrists, and they led the implementation of the mhGAP-IG at the pre-service level. Hence involving teaching psychiatrists in pre-service implementation of the mhGAP-IG could further facilitate its acceptance within the psychiatric community and strengthen its acceptance and implementation overall.

The study also demonstrates that the mhGAP-IG could be used both to develop new training programs and to strengthen and enhance existing curricula. It confirms that it is feasible to teach the mhGAP-IG to diverse groups of students. This again underscores the universal aspect of the mhGAP-IG tool, which can be used to build a common understanding among different categories of future health workers about the assessment and management of mental health disorders [12].

None of the academic institutions have stopped using the mhGAP-IG in their pre-service training, and some had been using it successfully for up to 3 years at the time of the study. This shows that using the mhGAP-IG can be a sustainable pre-service teaching strategy, despite challenges that may exist.

Resistance from some faculty members and a lack of support from institutional leaders were among the major obstacles to introducing the mhGAP-IG identified. These challenges were common, and so universities planning to introduce the mhGAP-IG should mitigate them in advance, in particular securing support from the leadership of the institution. Underlining this, the study reveals that support from university leadership was one of the most important facilitating factors in the implementation of the mhGAP-IG in pre-service training.

Another common challenge noted was insufficient time for teaching using the mhGAP-IG. This indicates that university departments were too optimistic about the time required to teach and study using mhGAP-IG tools. Those considering introducing the mhGAP-IG should think very carefully about the time and other resources needed and be ready to adjust time slots allocated to teach the mhGAP IG, as was done by UNAM in Mexico.

Students’ involvement in implementing the mhGAP-IG, support from decision makers, and providing more time for training with it were some of the most important recommendations for the successful introduction of the mhGAP-IG into pre-service training.

The study found that all the institutions taking part (except for Phebe Paramedical Training Program and School of Nursing in Liberia) have introduced and are using the mhGAP-IG without extra financial support. While no doubt additional funding would have helped to introduce the mhGAP-IG in pre-service settings, this also demonstrates that this kind of training is cost-effective as it can be implemented and sustained without additional funding. This is in contrast with in-service training, which has several cost implications attached, including per diem allowances to be paid to trainees, professional fees for facilitators, renting training venues, and so on [34, 35].

### Strengths and limitations of the study

This study includes academic institutions in different geographical regions and different multi-disciplinary settings. However, it has some limitations. The sampling technique chosen for the study was convenience sampling. The study could be subject to self-reporting bias as the questionnaire was self-administered. Also, as the time elapsed since the implementation of the mhGAP-IG varied from less than one year to more than 3 years, the study could be subject to recall bias when collecting information about the initial decision to use the mhGAP-IG and major obstacles, facilitating factors, and challenges encountered with pre-service training where it was used.

## Conclusions

This study indicates that mhGAP-IG pre-service training can be successfully implemented in diverse settings with different students (medical, nursing, doctors in training) and can be adapted to meet the unique needs of different institutions. mhGAP-IG pre-service training appears to be feasible and acceptable when implemented in a range of pre-service training settings.

## Data Availability

All data are given in the results section and in Table 1.

## Abbreviations

DALYs: Disability-adjusted life years
IACAPAP: International Association for Child and Adolescent Psychiatry and Allied Professions
KMU: Kyiv Medical University
KRSU: Kyrgyz-Russian Slavic University
LMICs: Low- and middle-income countries
MNS: Mental, neurological, and substance use disorders
mhGAP: Mental Health Gap Action Programme
mhGAP-IG: Mental Health Gap Action Programme Intervention Guide
NMAPE: P.L. Shupyk National Medical Academy of Postgraduate Education
TSMU: Tbilisi State Medical University
UOH: University of Hargeisa
UI: University of Ibadan
UNAM: National Autonomous University of Mexico
WHO: World Health Organization
WPA: World Psychiatric Association
YSMU: Yerevan State Medical University after Mkhitar Heratsi
